# Call for a consensual definition of dyslipidemia in coronary angiography trials

**DOI:** 10.3389/fcvm.2025.1506149

**Published:** 2025-02-05

**Authors:** Aurélien Clerc, Mario Togni, Stephane Cook

**Affiliations:** Cardiology, University and Hospital Fribourg, Fribourg, Switzerland

**Keywords:** dyslipidemia (DLP), coronary angiography (CA), cardiovascular risk factor, coronary artery disease, definition

## Abstract

Dyslipidemia is extensively analyzed in clinical trials investigating its role as a risk factor for coronary artery disease (CAD). However, its definition varies vastly among studies, leading to different attributions to the variable dyslipidemia. The objectives of this study are to verify the hypothesis of a lack of a consensual definition of dyslipidemia in coronary angiography studies and to propose a consensual definition of dyslipidemia, considering the influence of each serum lipid parameter on mortality. A systematic search of coronary angiography studies focusing on dyslipidemia was conducted. We listed definitions and their references in the 258 articles the research found. Out of the 258 articles retrieved in the search, 52 studies (20%) provided a definition of dyslipidemia, and 20 (8%) mentioned the source. We identified 39 different definitions. To mitigate misinterpretations of cardiovascular risk factors, we propose the use of the “lipid triad” components to define dyslipidemia: LDL-cholesterol >3.0 mmol/L for primary prevention and >2.6 mmol/L or >1.4 mmol/L for secondary prevention in patients over/under 75 years old, respectively; *or* HDL-cholesterol <1.3 mmol/L (women) and <1.0 mmol/L (men); *or* triglycerides >1.7 mmol/L.

## Introduction

Cardiovascular diseases stand as the primary cause of morbidity and mortality globally (1). Dyslipidemia is unequivocally recognized as a major cardiovascular risk factor according to the most cardiologic societies, such as the European Society of Cardiology (ESC), the European Atherosclerosis Society (EAS), the American College of Cardiology (ACC) or the American Heart Association (AHA) (2, 3). Moreover, it ranks among the most extensively studied, treated, and referenced factors in the scientific literature.

The Standardized Nomenclature of Medicine (SNOMED) defines dyslipidemia as a disorder of lipoprotein and/or a disorder of lipid metabolism (4). The Medical Subject Headings (MeSH) thesaurus produced by the US National Library of Medicine proposes the following definition of dyslipidemia: “Abnormalities in the serum levels of lipids, including overproduction or deficiency. Abnormal serum lipid profiles may include high total cholesterol (TC), high triglycerides (TG), low high density lipoprotein cholesterol (HDL-C), and elevated low density lipoprotein cholesterol (LDL-C)” (5). The most recent guidelines from ESC/AES and ACC/AHA lack a specific statement on the parameters and respective thresholds defining dyslipidemia (2, 3). They rather focus on cardiovascular risk management according to specific characteristics (age, concomitant risk factors, secondary prevention etc.). This risk management is often based on LDL-C reduction, necessarily influenced by statins (6). Anyway, these definitions are neither systematic nor unanimous, and highly influenced by pharmacology.

Thus, relying parameters of dyslipidemia to a relevant, global and only health outcome would offer an objective and unbiased perspective. In that sense, mortality seems to be the best catch to build a sound and universal definition.

In coronary angiography trials, dyslipidemia is often collected and analyzed to assess its predictive value or association with CAD. However, its definition varies, sometimes with or without cutoffs, and is barely referenced. One study might use lipid-lowering therapy, such as statins, as a criterion for dyslipidemia, while another may employ thresholds for one or more serum lipids. These discrepancies in defining dyslipidemia can yield different results. The identification of a participant as “dyslipidemia: yes” at >2 or >3 mmol/L LDL-C can significantly alter the number of participants tagged with or without dyslipidemia. Moreover, relying solely to treatment, given the poor adherence to statins (7), introduces potential biases. Before evaluating how discrepancies in defining dyslipidemia could impact results in clinical trials regarding its predictive role for CAD, it is essential to delineate the range of dyslipidemia definitions in such studies and gather their referential bases.

This paper aims to verify the hypothesis that there is a lack of a consensual and/or systematic definition of dyslipidemia in coronary angiography studies. The secondary objective is to propose a consensual definition of dyslipidemia, considering the influence of each serum lipid parameter on mortality.

## Materials & methods

To ascertain the true divergence in dyslipidemia definitions within coronary angiology studies, we meticulously cataloged the range of definitions found in a single database. Our systematic literature search on PubMed up to January 15, 2024 employed MeSH terms “coronary angiography” and “dyslipidemia” in the following query: ((coronary angiography[MeSH Terms]) OR (coronary angiography[Title/Abstract])) AND ((dyslip*[Title/Abstract]) OR (dyslip*[MeSH Terms])). It's noteworthy that we focused on one database, as our goal was not an exhaustive review but rather an exercise illustrating our point. The research query, crafted with MeSH terms and title/abstract criteria, aimed to identify studies specifically addressing the subject.

### Selection criteria

We chose to select the studies written in English that were published in the last five years, because a certain number of guidelines of the ESC/EAS and ACC/AHA were published for the last time in 2019 (8, 9). We then excluded case studies and those pertaining to familial hypercholesterolemia, which already have well-established criteria (8, 10, 11). The principal investigator then compiled a list of dyslipidemia definitions from the included articles.

### Data collection

In collecting data from these articles, we tabulated the definitions when explicitly stated. Additionally, we recorded the cited references accompanying these definitions. In cases where an article indicated that the dyslipidemia definition had been reported elsewhere or when the references were imprecise (e.g., without the year of the guidelines), we did not take it into account. If the reference was precise (e.g., 2019 ESC/EAS Guidelines for the management of dyslipidemias) but lacked specific values in the text, we considered the definition as referred.

### Illustrative case studies

To exemplify potential discrepancies, we considered two cases. Case #1 involved a middle-aged man with recent CAD, no prior lipid-lowering treatment, treated hypertension, a positive family history, and a history of former smoking. His lipid profile comprised total cholesterol (TC) of 4.8 mmol/L (185 mg/dl), LDL-cholesterol of 3.4 mmol/L (131 mg/dl), HDL of 1.04 mmol/L (40 mg/dl), and triglycerides (TG) of 1.3 mmol/L (115 mg/dl). Case #2 was a 68-year-old woman with recent CAD, no prior lipid-lowering treatment, and only treated hypertension as a cardiovascular risk factor. Her lipid profile comprised TC of 4.5 mmol/L (174 mg/dl), LDL-cholesterol of 3.02 mmol/L (116 mg/dl), HDL of 1.23 mmol/L (48 mg/dl), and TG of 1.14 mmol/L (101 mg/dl). We examined whether both cases met the criteria for “dyslipidemia: yes” or “dyslipidemia: no” based on the definitions proposed in the studies.

### Definition

Finally, in crafting the most widely accepted definition of dyslipidemia, we primarily considered the association between each serum lipid and mortality, which seems to be one of the most relevant and universal outcome. Indeed, mortality emerged as the most robust indicator for the general population, aligning with many guidelines using mortality or composite endpoints in studies on serum lipids. Moreover, we decided not to consider lipid-lowering therapies as a criterion of the consensual definition, since what matters is the amount of blood lipid particles and not the means to achieve them—which could also be lifestyle by the way. A recent meta-analysis indicated that lipid-lowering therapy (statins) had limited impact on absolute mortality and myocardial infarction rates (12). Focusing on mortality provides a more detached perspective from statin-focused studies. And we also decided not to consider history of dyslipidemia for the consensual definition, since it has largely been explained that definition of dyslipidemia was too heterogeneous yet. Hence, the definition of dyslipidemia will be crafted from the serum lipids only.

## Results

Our research, conducted with defined criteria, yielded 310 articles. After screening, we excluded 24 case reports, 13 studies related to familial hypercholesterolemia, and 15 non-English articles. This left us with 258 studies for the definition listing.

Of these 258 studies, 52 (20%) provided a definition for dyslipidemia. Within these 52 studies, we identified 39 distinct definitions. We excluded one study due to criteria specific to a transplant condition, resulting in 38 definitions from 51 studies summarized in Table 1. These definitions varied in scope, often involving abnormal levels of one serum lipid but not consistently so (e.g., only lipid-lowering therapy). Some were composite (e.g., TC >5.2 mmol/L AND TG >1.7 mmol/L), while others were not (e.g., TC >5.2 mmol/L OR TG >1.7 mmol/L OR LDL-C >3.6 mmol/L OR history OR treatment).

**Table 1:** Criteria characterizing the dyslipidemia in the 51 studies proposing a definition.

Criterion	Most frequently found	Range of values/diversity
High total cholesterol (TC)	TC level ≥5.2 mmol/L (200 mg/dl) or ≥6.2 mmol/L (240 mg/dl)	≥4.9–6.5 mmol/L (190–250 mg/dl) “increased”
High LDL-cholesterol (LDL-C)	LDL-C level ≥3.4 mmol/L (130 mg/dl), 3.6 mmol/L (140 mg/dl), 4.1 mmol/L (160 mg/dl)	≥1.4–4.9 mmol/L (55–190 mg/dl) “high”, “increased”, “over the goal defined by physicians”
Low HDL-cholesterol (HDL-C)	HDL-C ≤1.3 mmol/L (50 mg/dl) or ≤1 mmol/L (40 mg/dl) by women resp. men	≤.8–1.3 mmol/L (30–50 mg/dl) “low”, “reduced”
High triglycerides (TG)	TG ≥1.7 mmol/L (150 mg/dl)	1.7–2.8 mmol/L (150–250 mg/dl) “high”, “increased”, “elevated”
Treatment	Use of lipid-lowering therapy	
Diagnosis	Diagnosis present in the patient's record History of dyslipidemia	

Among the 258 studies, 20 (8%) referenced their dyslipidemia definition. Excluding one specific to liver transplant, we considered 19 articles, using 13 different references defining dyslipidemia, mostly US, European and Chinese Cardiovascular Societies' guidelines.

To illustrate the discrepancies in defining a subject as positive or negative for the variable “dyslipidemia”, we found that among 38 definitions (51 articles), case #1 would have been labeled “dyslipidemia: yes” in 14 definitions (19 articles) and “dyslipidemia: no” in 24 definitions (32 articles). Specifically, 6 articles attributed it to a low HDL-cholesterol level, 12 articles to an increased LDL-cholesterol level, and 1 article to the status, assuming a physician-defined lower LDL-cholesterol target based on formal smoking, hypertensive status, and positive family history. Case #2 would also have been labeled “dyslipidemia: yes” in 14 definitions (19 articles) and “dyslipidemia: no” in 24 definitions (32 articles). Specifically, 13 articles attributed it to a low HDL-cholesterol level and 6 articles to an increased LDL-cholesterol level.

The association between mortality and the serum lipids is summarized in Table 2. The ideal levels proposed are explicated in the discussion.

**Table 2:** Serum lipids and their association with mortality, aiming to define ideal values to craft a consensual definition of dyslipidemia.

Serum lipid	Nature of the association with mortality	Ideal value(s)
Total cholesterol (TC)	Overall: negative or U-shaped Cardiovascular: positive	*Depends on the LDL-C values*
LDL-cholesterol (LDL-C)	Overall: negative or U-shaped Cardiovascular: positive	<3 mmol/L (115 mg/dl) primary prevention <1.4 mmol/L (55 mg/dl) or 2.6 mmol/L (100 mg/dl) secondary prevention, age <75 resp. >75 years
HDL-cholesterol (HDL-C)	Negative	>1.3 mmol/L (50 mg/dl) women >1 mmol/L (40 mg/dl) men
Triglycerides (TG)	Mostly inconclusive	<1.7 mmol/L (150 mg/dl)

## Discussion

In this critical analysis of dyslipidemia categorization in clinical trials, our primary objective was to investigate the hypothesis of a lacking consensus in the definition of dyslipidemia. Among the 258 studies included, we found that 80% did not provide a definition of dyslipidemia. Furthermore, of the studies that did specify dyslipidemia classification, we identified 38 different definitions. The lack of references justifying these definitions was also notable, indicating a lack of consensus in criteria across clinical trials.

Dyslipidemia poses a unique challenge in consensus definition compared to other well-established cardiovascular risk factors such as hypertension or smoking. Unlike conditions with clear definitions, dyslipidemia often lacks explicit criteria in studies, as evidenced by the work of Senoner and colleagues (13), for instance, where a clear definition of every cardiovascular risk factor but dyslipidemia was stated.

Several factors contribute to the complexity and lack of consensus in defining dyslipidemia. Firstly, dyslipidemia is not solely characterized by a single criterion (e.g., total cholesterol (TC), LDL-cholesterol (LDL-C), HDL-cholesterol (HDL-C), triglycerides (TG)), making it a multivariable equation. Unlike other cardiovascular risk factors such as arterial hypertension, diabetes mellitus, or smoking status, dyslipidemia is more complex to define. Secondly, the modifiability of dyslipidemia parameters varies. LDL-C and TC are effectively reducible through lipid-lowering treatments, while HDL-C and TG respond more to lifestyle changes (14, 15). TG levels are influenced by factors such as diet, alcohol consumption, and body weight (15–18). HDL-C is sensitive to physical activity and body weight, and to a lesser extent, diet and alcohol consumption (15, 19). The multitude of factors influencing dyslipidemia, combined with the lack of homogeneity across sex, ethnicity, country, and age (20–23) contributes to the difficulty in establishing a consensus definition.

Despite these challenges, having a common definition, similar to hypertension or diabetes, would reduce confusion, as demonstrated in the illustrative examples above.

Few studies have comprehensively compared all serum lipids within the same study. Lower concentrations of TC, LDL-C, and TG, coupled with elevated HDL-C, are mainly associated with mortality, but not consistently with cardiovascular disease (CVD) mortality in men and women (24, 25). Heterogeneous findings suggest the influence of significant confounding factors (heredity, age, diet, physical activity) (21, 26). For example, the Framingham cohort taught that targeting optimally low TC and LDL-C is not efficient in terms of survival, particularly as individuals age (21). A Thai study and a recent meta-analysis asserted that among serum cholesterol parameters, HDL-C has the strongest inverse association with CVD mortality (27, 28).

TC is particularly tested in studies prior to 2000, because it was the more readily available. For overall and non-CVD mortalities, the association with TC is mainly negative or U-shaped (22–24, 29, 30). The hypothesis for the inverse association between TC and overall mortality would be that a confounding factor, such as unintentional weight loss, induces lower TC and premature death (31–33). The association between TC and CVD mortality is mostly positive in different contexts (country, ethnic group, age…), but particularly marked in white men below 60–70 years old (20–22, 28, 34–36), probably because their number was the highest in the studies. So defining an ideal level of TC should depend on age and cardiovascular history. And as long as the main part of TC is LDL-C (37), stop considering TC level to define dyslipidemia seems rational, in order to make the easiest possible consensual definition of dyslipidemia. Moreover, unlike in the 20th century, LDL-C is nowadays highly available.

LDL-C follows logically the same association with mortality as TC. A large number of studies comprising a systematic review found an inverse or U-shaped association between LDL-C and overall mortality (21, 38–40). The same results were found for women but usually in a weaker way (41–43). As for TC, this not instinctive inverse association between LDL-C and overall mortality is certainly to connect with malnutrition (44), but also with reduced cholesterol synthesis capacity when aging (45). A positive or U-shaped association between LDL-C and CVD mortality is usually reported, particularly in men and young subjects (28, 42). An ideal one-size-fits-all LDL-C level in the general population is not yet defined (46), unlike in secondary prevention, where decreasing LDL-C level is highly effective to prevent a new cardiovascular event (8). A Danish study found the lowest risk of all-cause mortality at LDL-C level of 3.6 mmol/L (140 mg/dl) in the general population and 2.3 mmol/L in the cardiovascular secondary prevention population (39). Thus, like TC, considering at least age and cardiovascular history seems of paramount importance in defining an ideal LDL-C level. The level of evidence is now sufficient to assert that patients below 75 years old with a CVD history benefit from an LDL-C level <1.4 mmol/L (55 mg/dl) (6, 8). For those subjects, an LDL-C above 1.4 mmol/L could be considered dyslipidemia. For subjects over 75 years old with a CVD history, evidence for a targeted LDL-C level is still controversial (8, 47). While reducing LDL-C level to 1.4, 1.8, or 2.2 mmol/L (55, 70, resp. 84 mg/dl) does not seem to be efficient in reducing overall mortality in older patients (43, 48–50) according to the diminished capacity to produce cholesterol and the harmful observed effect of lowering LDL-C, the objective of 2.6 mmol/L (100 mg/dl) was qualified as not efficient enough (51), even if it was the goal 20 years ago (52). So, as stated by Lucchi, moderation in the LDL-C target for secondary cardiovascular prevention in oldest people appears to be the soundest option. The threshold of 2.6 mmol/L could thus be suggested as a cutoff for the qualification of dyslipidemia in older patients in cardiovascular secondary prevention. For subjects without CVD history, a LDL-C up to 3 mmol/L (115 mg/dl) does not correspond to a marked risk (8).

A low HDL-C level is associated with mortality, particularly CVD mortality. The association is often U-shaped (24, 53–57) and specifically strong in younger subjects (58–60). But compared to low TC and LDL-C, this protective effect of a higher HDL-C seems to stay longer throughout life (26, 59). Some studies have tested the effect of alcohol intake and it appeared that when HDL-C was increased because of alcohol intake, its protective effect was weaker (57, 61). Inversely, physical activity seems to be a convincing enhancing factor that reinforces the protective effect of HDL-C on mortality (62, 63). It appears that the inverse association between HDL-C and mortality – particularly CVD mortality – is the clearest among serum lipids (27, 28). But as long as increasing HDL-C do not lower cardiovascular events (8, 64), the association could be mediated by a confounding factor like diet or physical activity. The ideal level of HDL-C seems to be >1.3 mmol/L for women (50 mg/dl) and >1 mmol/L (40 mg/dl) for men (53–56).

The association between TG and mortality is little tested and when tested, the association is mostly inconclusive (27, 34, 65, 66), although a recent Korean prospective study found a clear positive association between TG levels and CVD mortality (67). As mentioned above, TG level is notably influenced by diet, alcohol consumption and glycemic control. An ideal level of TG is pretty consensual among guidelines at <1.7 mmol/L (150 mg/dl) (8, 68, 69).

In summary, we propose the following criteria for labeling a subject as “dyslipidemia: yes”:

- LDL-C >3 mmol/L (115 mg/dl) (except for people with CVD history for whom a LDL-C >1.4 mmol/L (55 mg/dl) or >2.6 mmol/L (100 mg/dl) should be considered as too high for subjects under resp. over 75 years old) *or*
- HDL-C <1.3 mmol/L for women (50 mg/dl) and <1 mmol/L (40 mg/dl) for men *or*
- TG >1.7 mmol/L (150 mg/dl).

These three serum lipids collectively form the “lipid triad” due to atherogenic power (37, 70, 71).

In our proposed definition, lipid-lowering treatment and a “history” of dyslipidemia are not considered criteria, as the focus is on current lipid levels. Contrary to some studies, these two characteristics are not deemed sound criteria for defining dyslipidemia. Nevertheless, we strongly suggest collecting data on lipid-lowering treatment as a separate variable.

This study critically analyzed dyslipidemia characterization in coronary angiography trials, representing – to our knowledge – the first to undertake such an exercise. While limited to a single scientific database, albeit the most widely used, extending to other databases may further enrich observations. The focus on clinical trials in coronary angiography, while a strength, means the study only covers one facet of cardiovascular research. Broadening the search to encompass all cardiovascular studies would offer a more comprehensive understanding.

## Conclusions

This study highlights significant heterogeneity in dyslipidemia characterization within clinical trials, which could potentially lead to misinterpretations of cardiovascular risk factors for coronary artery disease. Proposing a consensual definition based on mortality aims to uniformly classify subjects as positive or negative for this common condition. The proposed definition, derived from numerous studies, provides a more robust basis for data accumulation and comparison within studies.
